# Water‐Group Pickup Ions From Europa‐Genic Neutrals Orbiting Jupiter

**DOI:** 10.1029/2022GL098111

**Published:** 2022-05-04

**Authors:** J. R. Szalay, H. T. Smith, E. J. Zirnstein, D. J. McComas, L. J. Begley, F. Bagenal, P. A. Delamere, R. J. Wilson, P. W. Valek, A. R. Poppe, Q. Nénon, F. Allegrini, R. W. Ebert, S. J. Bolton

**Affiliations:** ^1^ Department of Astrophysical Sciences Princeton University Princeton NJ USA; ^2^ The Johns Hopkins University Applied Physics Laboratory Laurel MD USA; ^3^ Laboratory for Atmospheric and Space Physics University of Colorado Boulder Boulder CO USA; ^4^ Geophysical Institute University of Alaska Fairbanks Fairbanks AK USA; ^5^ Southwest Research Institute San Antonio TX USA; ^6^ Space Sciences Laboratory University of California Berkeley CA USA; ^7^ Institut de Recherche en Astrophysique et Planétologie CNRS‐UPS‐CNES Toulouse France; ^8^ Department of Physics and Astronomy University of Texas at San Antonio San Antonio TX USA

## Abstract

Water‐group gas continuously escapes from Jupiter's icy moons to form co‐orbiting populations of particles or neutral toroidal clouds. These clouds provide insights into their source moons as they reveal loss processes and compositions of their parent bodies, alter local plasma composition, and act as sources and sinks for magnetospheric particles. We report the first observations of H_2_^+^ pickup ions in Jupiter's magnetosphere from 13 to 18 Jovian radii and find a density ratio of H_2_^+^/H^+^ = 8 ± 4%, confirming the presence of a neutral H_2_ toroidal cloud. Pickup ion densities monotonically decrease radially beyond 13 *R*_J_ consistent with an advecting Europa‐genic toroidal cloud source. From these observations, we derive a total H_2_ neutral loss rate from Europa of 1.2 ± 0.7 kg s^−1^. This provides the most direct estimate of Europa's H_2_ neutral loss rate to date and underscores the importance of both ion composition and neutral toroidal clouds in understanding satellite‐magnetosphere interactions.

## Key Points

- First identification of H_2_^+^ in the Jovian magnetosphere from 13 to 18 *R*_J_, with a density ratio of H_2_^+^/H^+^ = 8 ± 4%
- H_2_^+^ in Jupiter's magnetosphere is predominantly produced by Europa's neutral toroidal cloud
- Europa's total H_2_ neutral loss rate is 1.2 ± 0.7 kg s^−1^

## 1 Introduction

Each of the outer three Galilean satellites Europa, Ganymede, and Callisto have surficial ice and atmospheric water group species (H, H_2_, O, OH, H_2_O, and O_2_). These species are abundant to varying degrees due to differing surface compositions and atmospheric source/loss processes (e.g., Ip, 1996; Johnson, 1990, 2004; Marconi, 2007; McGrath et al., 2004; Roth, 2021; Saur et al., 1998; Smyth & Marconi, 2006), where atmospheric losses could sustain neutral toroidal clouds. Of these three satellites, only Europa's neutral toroidal cloud has been inferred from observations (Lagg et al., 2003; Mauk et al., 2003) and is assumed to be produced by sublimation and sputtering from its icy surface material (Smyth & Marconi, 2006). Depending on the lifetimes of orbiting neutrals, these clouds may or may not completely populate all longitudes throughout their parent body's orbits. Hence, we refer to them as “neutral toroidal clouds” in lieu of the historically common “neutral tori” terminology.

As observed by the Galileo spacecraft, energetic neutral atoms originating just outside Europa's orbit, born via charge exchange between a cold neutral toroidal cloud and energetic charged particle environment, suggest the densities in Europa's neutral gas toroidal cloud are comparable to Io's neutral gas toroidal cloud (Mauk et al., 2003, 2020). Unlike near Io's orbit, however, where the dense Io plasma population quickly ionizes neutrals, the less dense plasma environment at Europa and beyond is more conducive to longer neutral lifetimes (Bagenal & Dols, 2020). Separately, observations of depletions in energetic protons that would primarily reside at low magnetic latitudes (pitch angles near 90°) were proposed to be due to charge exchange with the very same neutral Europa toroidal cloud (Lagg et al., 2003). Neither observation was able to directly determine the composition of the neutral cloud, however, ultraviolet observations ruled out a dominantly oxygen‐rich cloud (Hansen et al., 2005). Europa's neutral toroidal cloud was subsequently modeled using multiple atmospheric constraints, and a cloud predominantly composed was proposed to explain both indirect cloud observations (Smith et al., 2019; Smyth & Marconi, 2006). H_2_ is understood to have the highest escape rate because of its enhanced surface abundance from radiolysis, large‐scale height, light mass, and non‐condensability as a diatomic species from Europa (Smyth & Marconi, 2006), Ganymede (Marconi, 2007), and Callisto (Mogan et al., 2021).

While remote observations did not detect an oxygen‐rich toroidal cloud and subsequent modeling efforts have suggested a dominantly H_2_‐rich Europa cloud, H_2_ had yet to be definitively measured and direct constraints on its abundances or loss rates were not possible given the prior observations. Here, we present direct observations of H_2_^+^ pickup ions sourced from a neutral H_2_ toroidal cloud in the Europa‐Ganymede region. We use these observations to constrain the total abundances and loss rate of neutral H_2_ from Europa. In Section 2, we discuss how and where H_2_^+^ pickup ions are observed from the Juno spacecraft. In Section 3, we use these observations to determine numerical densities of both H^+^ and H_2_^+^ and compare these to a plasma advection model to estimate loss rates from Europa in Section 4. We conclude in Section 5 with a discussion on implications for loss rates from the other Galilean satellites and future work that could build on these results.

## 2 Observations of H_2_^+^

The Juno mission (Bolton et al., 2017) carries the Jovian Auroral Distributions Experiment (JADE; McComas et al., 2017), a suite of plasma instruments that includes an ion time‐of‐flight (TOF) mass spectrometer. JADE's ion sensor measures the flux of ions with energy‐per‐charge of 10 eV/Q to 46 keV/Q and a mass‐per‐charge of ∼1–64 AMU/Q. Juno's eccentric, nearly polar orbit precesses such that its furthest distance (apojove) is sequentially more southward after each close approach (perijove). Therefore, the location where Juno intersects Jupiter's equatorial plane moves inward each orbit, enabling ion composition observations by JADE's TOF mass‐spectrometry of the equatorial plasma environments at varying radial distances (Kim et al., 2020a, 2020b).

By late‐2021 up to and including its 37th perijove, Juno transited the equatorial plasma environments at the orbital distances of Europa, Ganymede, and Callisto. A JADE signal consistent with H_2_^+^ is present near the equatorial region inside ∼20 *R*_J_ (1 R_J_ ≡ 71,492 km). Isolating H_2_^+^ in the JADE data set requires removing multiple background and foreground sources that overlap with the H_2_^+^ signal in the combined TOF versus energy space. Notably, the H_2_^+^ feature overlaps with H^+^ in this space, where H^+^ is more abundant, hence correctly subtracting the proton foregrounds is critical. We apply three background subtractions, and additionally remove the signature of H^+^ foreground to isolate the count rates due to H_2_^+^. To remove the H^+^ signal, we use an empirical response from a unique event where Juno was connected to Io's Main Alfvén Wing (Szalay et al., 2020). During this period, large proton abundances were observed and energized throughout the entire observable range across multiple Juno instruments (Clark et al., 2020; Sulaiman et al., 2020) enabling a reliable determination of the in‐flight response to protons alone. The subtraction schemes are described in detail in Supporting Information S1.

We manually identified regions where H_2_^+^ could be reasonably separated, producing a conservative subset of JADE data with high‐fidelity H_2_^+^ data. In the region past 10 *R*_J_ near the magnetic equator, an unambiguous H_2_^+^ signature is observed from ∼13 to 18 *R*_J_. Outside these locations, H_2_^+^ signatures are still observed, but cannot be fully separated from foregrounds and backgrounds with the current techniques.

**Figure 1:**
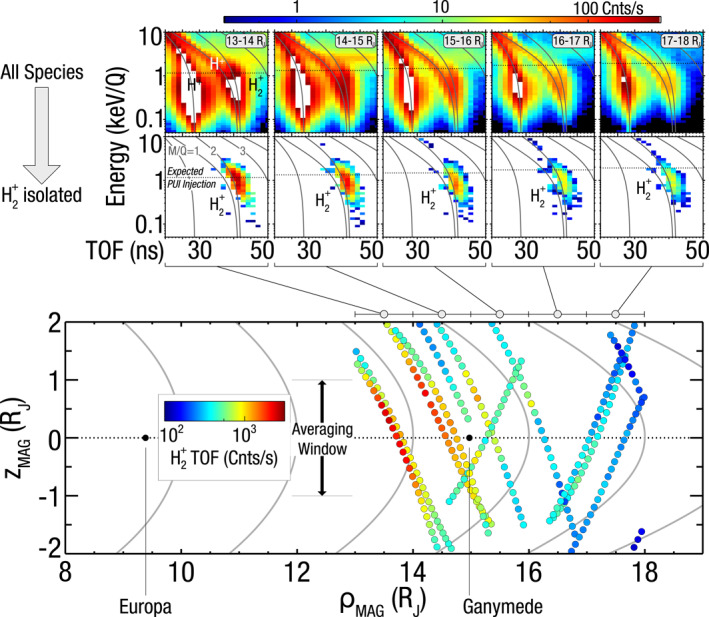
Count rates of H_2_^+^ from 13 to 18 *R*_J_ (top) Ion energy per charge as a function of time‐of‐flight (TOF) for all species with nominal background subtraction (upper row) and with H_2_^+^ isolated (lower row) for all observations within an averaging window of ±1 *R*_J_ from the magnetic equator. H_2_^+^ is unambiguously observed when foregrounds and backgrounds are removed, in the vicinity of the second gray TOF trace for a mass per charge of 2. The horizontal line indicates the expected detected energy for a freshly created pickup ion at the spacecraft's position (bottom) Locations of each observation in cylindrical coordinates with the +*z* axis are aligned with Jupiter's magnetic dipole. H_2_^+^ is observed with the strongest magnitude in the inner‐most detected locations.

Figure 1 shows these H_2_^+^ TOF observations, where each of the top TOF spectra are the superposed average count rate spectra within ±1 *R*_J_ from the magnetic equator for each 1 *R*_J_ radial bin shown in the bottom panel. Times used in these combined TOF spectrograms are given in Table S1 in Supporting Information S1. Without background and foreground H^+^ subtraction, the counts along the *M*/*Q* = 2 track are dominated by H^+^ and to varying degrees, a long TOF tail from heavy ions that manifests as a horizontal bar around 2–4 keV notable in the second TOF spectrogram (14–15 R_J_). As indicated in Figure 1, which shows the expected energy and TOF tracks for different species, protons are detected along two separate tracks due to the internal workings of the instrument. See Text S1 in Supporting Information S1 for additional description on how this is accounted for in the H_2_^+^ identification scheme.

The H_2_^+^ count rates occur near and mostly below the expected local corotation energy (Text S2 in Supporting Information S1), consistent with a population of pickup ions corotating with Jupiter's magnetosphere sourced from a neutral gas moving slowly with respect to local corotation speed. Pickup ion fluxes are expected to peak in the detected energy range of 100s eV to a few keV when Juno is within 10–30 *R*_J_. Therefore, the instrument's energy range of 10 eV/Q–46 keV/Q is sufficient to capture any pickup ions generated in this region. The bottom panel of Figure 1 shows where these observations occurred along with magnetic field lines every 2 *R*_J_ in equatorial distance using the JRM09 + CAN models (Connerney et al., 1981, 2018). As shown here, the largest H_2_^+^ count rates are observed near the magnetic equator and closest to Jupiter over this distance range.

## 3 Numerical Densities

While the TOF data set does not have any information on directionality, JADE observed the full‐sky over each ∼30s spin and we can calculate a partial numerical density from the count rates as a function of energy over the JADE energy bandpass. After all foregrounds and backgrounds are subtracted, we sum count rates over all TOFs corresponding to AMU/Q between 1.5 and 2.5 to determine a total count rate *R*_*obs*_ as a function of energy. JADE instantaneously observes an angular range of 270° extending from the anti‐sunward spin axis, such that for each spacecraft rotation it records counts from a total angular extent of 6π sr, double‐counting half the sky. We must reduce *R*_*obs*_ by an appropriate factor to determine the “true” average count rate *R = ηR*_*obs*_ corresponding to the 4π sr full sky (Text S3 in Supporting Information S1). Due to the instrument mounting and orbit geometry, for each H_2_^+^ observation by JADE, plasma corotating with Jupiter is predominantly observed on the hemisphere where JADE double‐counts incident populations, which also gives improved counting statistics. Table S1 in Supporting Information S1 gives values of *η*; for nearly all data analyzed here, *η* ≈ 0.5.

We convert count rate *R* into phase space density *f* via $f\left(v\right)=R\;/\;\left(G_{eff}^{v}\left(v\right)v^{4}\right)$ where $G_{eff}^{v}=\;G_{eff}^{E}/2$ is the energy dependent geometric factor (Kim et al., 2020a), with a factor of two between the energy geometric factor (e.g., McComas et al., 2021) and velocity geometric factor, and *v* is the measured energy per charge converted to speed for a molecule weighing two proton masses. In turn, the number density derived from a one‐dimensional phase space density is $n_{num}=4\pi \int_{0}^{\infty }f\left(v\right)v^{2}dv$. For JADE data with count rates in discrete energy bins, the numerical partial number density is given by $n_{num}=\;\frac{4\pi }{3}\underset{i}{\sum }\left(v_{i,\text{max}}^{3}-v_{i,\text{min}}^{3}\right)f\left(v_{i}\right)$, where *i* is the energy bin and each energy bin spans from *v*_*i,*min_ to *v*_*i,*max_ in velocity space and *v*_*i,*max_ *= v*_*i+1,*min_. We calculate numerical densities below 10 keV/Q to avoid additional backgrounds above this energy. This energy range is broad enough to well‐capture the H_2_^+^ features analyzed in this study. Finally, we must scale the numerical densities by a correction factor $n=\;\varepsilon n_{num}$ to account for a minority portion of H_2_^+^ counts in TOF × E space that are over‐subtracted in the vicinity of the H^+^ fork. See Text S1 in Supporting Information S1 and Kim et al. (2020a) for additional description on the proton “fork.” Text S4 in Supporting Information S1 describes this scaling factor, where we use a range of *ε* = 1.0, corresponding to no over‐subtraction, to *ε* = 1.6, a conservative upper bound on the correction factor.

**Figure 2:**
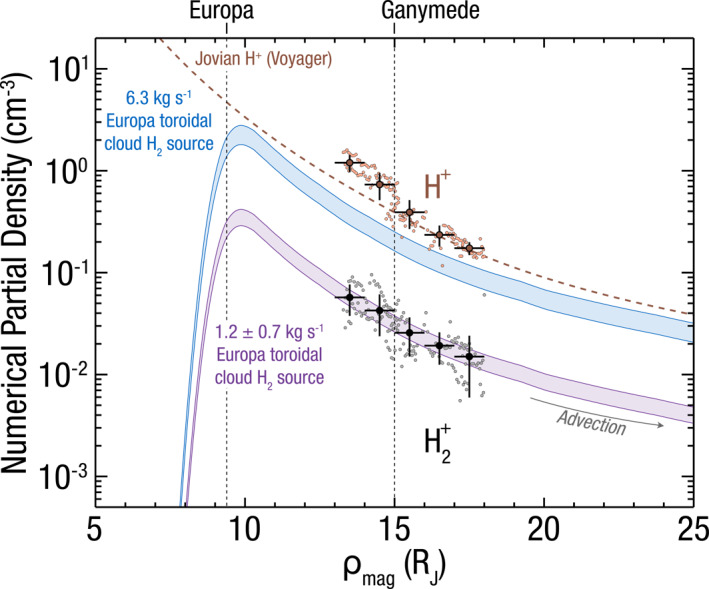
Partial density of H_2_^+^ pickup ions (gray) and H^+^ (orange), with average values every 1 R_J_ for *ε* = 1.2. The blue curves show expected H_2_^+^ pickup ion densities for a neutral Europa loss H_2_ rate of 6.3 kg s^−1^ (Smyth & Marconi, 2006). The purple curves show the best fit solution to the observed H_2_^+^ partial densities. These observations are consistent with a Europa neutral H_2_ loss rate of 1.2 ± 0.7 kg s^−1^. The orange dashed line shows an empirical model for proton densities based on Voyager observations (Bodisch et al., 2017).

Figure 2 shows these numerical densities for H^+^ and H_2_^+^ along with average values every 1 R_J_ for *ε* = 1.2. We include the H^+^ numerical densities to compare the method to model expectations, as there are no published estimates for the densities of H_2_^+^ pickup ions in the Jovian magnetosphere. The dark orange dashed line in Figure 2 shows the average expected Jovian proton density from an empirical model based on a reanalysis of Voyager data (Bodisch et al., 2017). Numerical densities for H^+^ are commensurate with previous JADE TOF‐derived densities in this region (Kim et al., 2020), and are also consistent with empirical model results for protons, lending confidence that the same numerical method applied on H_2_^+^ yields accurate estimates of the partial densities in these locations.

Using these methods, we find the H_2_^+^ ion densities monotonically decrease from 13 to 18 RJ, where H_2_^+^/H^+^ = 8 ± 4% for *ε* = 1.0–1.6. Such a low density ratio is consistent with the lack of detection by Voyager or Galileo plasma observations, which did not have the sensitivity or TOF mass‐spectroscopy capabilities that JADE has. We do not see any appreciable enhancement in the vicinity of Ganymede's orbit, although we did not include data taken very near to Ganymede itself. In the next section, we compare these densities with expectations from pickup ions generated by Europa's neutral toroidal cloud.

## 4 Comparison With a Europa Toroidal Cloud Source

For an inclusive range of plasma and solar ultraviolet conditions near Europa's orbit, electron impact ionization of H_2_ neutrals, $H_{2}+e^{-}\to H_{2}^{+}+2e^{-}$, is expected to be the dominant loss process for neutral H_2_ (Smith et al., 2019). H_2_^+^ pickup ions generated by this process will be radially transported outwards. The integrated transport time from 6 to 10 R_J_ is 10–40 days, while the transport time from 10 R_J_ to the outer magnetosphere (∼50 R_J_) is on the order of a few days with radial outflow speeds approaching 10s of km s^−1^ (Bagenal & Delamere, 2011). Additionally, a reanalysis of Galileo plasma data from PLS showed the radial gradient of flux tube content beyond 10 R_J_ is significantly reduced, precluding significant diffusion (Bagenal et al., 2016). Hence, we follow the assertion that while slow diffusive transport is valid in the inner magnetosphere (<10 R_J_), transport in the middle magnetosphere is better modeled with advection (Ng et al., 2018).

At these low magnetic latitudes, we assume H_2_^+^ pickup ions are transported radially outward via cylindrically symmetric advection. From conservation of mass, we solve the 1D time‐independent continuity equation in cylindrical coordinates $\frac{1}{\rho }\frac{\partial \left(n\rho v_{\rho }\right)}{\partial \rho }=P$, where *ρ* is cylindrical radial distance, *n* is the number density of H_2_^+^, *v*_*ρ*_ is the radial transport speed, and *P* is the production rate of pickup ions. This is solved using explicit finite differencing via $F_{i+1}=F_{i}+P_{i}\rho_{i}\Delta \rho$, where $F=\;n\rho v_{\rho }$, and a small value of Δρ=0.025 R_J_ is chosen such that the results are insensitive to the exact value of radial step size. We use a radial transport speed profile (Bagenal & Delamere, 2011) corresponding to a total Jovian plasma source of 0.7 ton/s, which represents the typical magnetospheric configuration (Delamere et al., 2004; Nerney & Bagenal, 2020). For the pickup ion input source, we approximate the modeled H_2_^+^ pickup ion input given in Figure 10 of Smyth and Marconi (2006) as $P_{E}\left(R\right)=P_{0E}e^{-\frac{{\left(R-R_{E}\right)}^{2}}{2\sigma_{E}}}$where *P*_*0E*_ is the production rate of H_2_^+^ and *R*_*E*_ = 9.4 R_J_, and σ_E_ = 0.4 R_J_. We assume transport occurs within a vertical extent of 2 R_J_.

To link the production rate of H_2_^+^ pickup ions to neutral H_2_ loss from Europa, we use existing estimates that 50%–77% of neutral H_2_ in the toroidal cloud is lost to H_2_^+^ pickup ions (Smith et al., 2019). The purple curves in Figure 2 show the best fit advection solutions to the binned H_2_^+^ data. Blue curves show expectations for the initial estimates of H_2_^+^ production, discussed in the following section. As shown here, the data are consistent with an advecting Europa‐genic toroidal cloud source of H_2_^+^ with a monotonically decreasing radial density profile past ∼13 R_J_. These fits are consistent with a total H_2_^+^ pickup ion production rate of 0.7 ± 0.3 kg s^−1^ for the full range of *ε* = 1.0–1.6. Hence, dividing this range by 0.5–0.77, a neutral H_2_ production rate within a 1‐sigma range of 1.2 ± 0.7 kg s^−1^ from the Europa toroidal cloud, lost from Europa's surface, is derived from the H_2_^+^ pickup densities observed. Table 1 gives these estimates along with those in the literature to date.

**Table 1:** Estimates for the Rate of Neutral H_2_ Mass Loss (kg s^−1^) and Number Loss (s^−1^) From Europa

H_2_ Loss (kg s^−1^)	H_2_ Loss (s^−1^)	Method	Reference
6.3	1.9 × 10^27^	Sputtering and atmosphere model	Smyth and Marconi (2006)
1.5	4.6 × 10^26^	Sputtering model	Plainaki et al. (2012)
0.7	2 × 10^26^	Sputtering model	Cassidy et al. (2013)
1.9	5.7 × 10^26^	Plasma‐atmosphere charge exchange model	Dols et al. (2016)
1.8	5.5 × 10^26^	Sputtering and atmosphere model	Vorburger and Wurz (2018)
1.2 ± 0.7	3.6 ± 2 × 10^26^	Pickup ion observations and advection model	This work

## 5 Discussion and Conclusions

**Figure 3:**
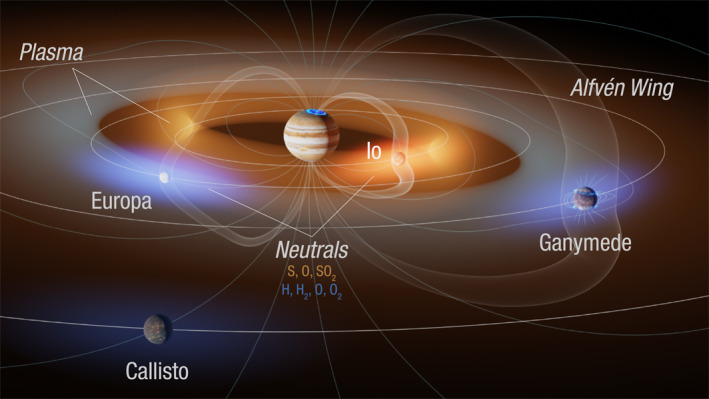
Overview of Jupiter's magnetosphere in the vicinity of the Galilean satellites. H_2_^+^ pickup ions (blue) originate from Europa's neutral toroidal cloud (brighter blue near Europa). Io and Europa contribute plasma pickup ions of different compositions to Jupiter's magnetosphere. Alfvén wings connected to the moons due to their interaction with corotating plasma are also shown in gray.

Using plasma observations from the JADE instrument onboard Juno, we detected a persistent population of H_2_^+^ pickup ions in Jupiter's magnetosphere near the magnetic equator from 13 to 18 R_J_. Using 1D numerical moments, we determine densities of H_2_^+^ to be in the range of 5 × 10^−3^ to 1 × 10^−1^ cm^−3^ throughout this region. The densities monotonically decrease as a function of radial distance and do not show any clear enhancement near Ganymede's orbit. The overall radial profile and total densities are consistent with pickup ions generated by a Europa‐genic neutral toroidal cloud with a loss rate of H_2_ from Europa of 1.2 ± 0.7 kg s^−1^. Hence, these observations confirm the existence and composition of Europa's neutral H_2_ toroidal cloud, which is expected to be the dominant component (Smith et al., 2019). Figure 3 shows a summary of the Jovian magnetosphere highlighting the existence of H_2_ neutrals and H_2_^+^ pickup ions associated with Europa.

The total loss rate of neutral H_2_ from Europa estimated here is a factor of 3–13 lower than the original estimated value of 6.3 kg s^−1^ (Smyth & Marconi, 2006), where the blue curves in Figure 2 show corresponding H_2_^+^ densities for this estimate. This comparison also shows that were the source rates from Europa to be this large, H_2_^+^/H^+^ ≳ 30% such that H_2_^+^ pickup densities would nearly rival those of protons in the magnetosphere. This is certainly not the case as previous measurements would have identified such a population (Voyager, Galileo, Juno, and New Horizons), and we find the density ratio to be H_2_^+^/H^+^ = 8 ± 4%.

While we derive our estimates from direct observations of the expected primary loss mechanism for Europa's neutral toroidal cloud (pickup ions), the values calculated in this work of 0.5–1.9 kg s^−1^ are commensurate with more recent estimates of 0.7–1.9 kg s^−1^ (Cassidy et al., 2013; Dols et al., 2016; Plainaki et al., 2012; Vorburger & Wurz, 2018) based on constraints of the loss rate from the surface due to sputtering inputs (Table 1). Both the sputtering estimates and our pickup ion calculations here require a synthesis of data and modeling to derive estimates; however, given that the two very different methods of estimating Europa's neutral H_2_ loss rate yield similar results, we can be reasonably confident that the loss rate is on the order of ∼1 kg s^−1^.

We refrain from estimating neutral H_2_ densities. Linking pickup ion densities to neutral cloud densities requires an additional set of model‐dependent assumptions above those already used in this analysis. Namely, the absolute lifetime of the H_2_ neutrals needs to be estimated to constrain neutral densities and doing so would incorporate additional model‐dependent uncertainty. Constraining neutral toroidal cloud densities using accurate lifetime estimates requires more complex modeling and is suggested as a future line of study.

Protons are also produced as pickup ions from Europa's neutral toroidal cloud, where the production rate of H^+^ is expected to be ∼15 times lower than that for H_2_^+^ (Smyth & Marconi, 2006). Therefore, we anticipate an input rate of protons to the Jovian magnetosphere from Europa to be less than 0.1 kg s^−1^. This value is at least an order of magnitude less input than the 2.5–13 kg s^−1^ required to sustain magnetospheric proton abundances inside 30 R_J_ (Bodisch et al., 2017), further pointing to Jupiter as the likely source for magnetospheric protons, where Juno observations have shown proton outflow contributes at least 1–5 kg s^−1^ to the magnetosphere (Szalay et al., 2021).

The largest source of uncertainty in our analysis lies in the use of a scaling factor to correct for over‐subtraction of H_2_^+^ counts (Text S4 in Supporting Information S1). Our conservative range of scaling factors leads to a large error bar in H_2_ mass loss from Europa that could be narrowed with improved methods to isolate H_2_^+^ count rates in JADE measurements. We also do not estimate densities in the vicinity of Europa's orbit in this study. Inside 13 RJ, particularly near Europa's orbit, two issues make measuring H_2_^+^ challenging. First, JADE experiences particularly large fluxes of penetrating radiation, adding to the background that must be removed. Second, and more importantly, locally produced pickup ions are expected to be observed around 0.5 keV and below, where the overlap between the H^+^ “fork” in TOF and H_2_^+^ in the JADE TOF data is particularly pronounced and *ε* may be significantly larger near Europa using the current method. Due to these reasons, we do not include observations in this region; however, future efforts involving forward‐modeling and/or fitting may help reveal the H_2_^+^ content in this region.

Ganymede is also a source of neutrals (Huang & Siscoe, 1987) and like Europa, is expected to predominantly shed H_2_ to generate a population of co‐orbiting neutrals (Marconi, 2007). However, such a neutral source would be more dispersed in a larger total volume as the moon orbits at 15 R_J_ compared to Europa's 9.4 R_J_ distance. Additionally, the radial transport speed is approximately an order of magnitude higher at 15 R_J_ compared to 9.4 R_J_ (Bagenal & Delamere, 2011), leading to shorter pickup ion lifetimes and densities. Hence even with equal neutral loss source rates from the two bodies, lower densities of neutrals and significantly lower peak densities of subsequent pickup ions would be expected near each of their orbits. While the monotonically decreasing densities as a function of radial distance are entirely consistent with a single source from Europa, we can very roughly constrain the contribution from Ganymede. Assuming Ganymede's neutral toroidal cloud has the same pickup ion profile $P_{G}\left(R\right)=P_{0G}e^{-\frac{{\left(R-R_{G}\right)}^{2}}{2\sigma_{G}}}$, where *R*_*G*_ = 15.0 R_J_ and σ_R_ = σ_E_ = 0.4 *R*_J_, we find source rates above ∼50% of Europa's would present a detectable departure from the approximately power‐law‐like radial profile observed for H_2_^+^, and therefore do not expect Ganymede's toroidal cloud H_2_^+^ pickup ion production rate to be larger than 50% of Europa's. If the conversion of H_2_ neutrals to H_2_^+^ pickup ions near Ganymede's orbit is similar to Europa's orbit, H_2_ neutral production at Ganymede would also be lower than ∼50% of Europa's. This effect is even more dramatic for Callisto at 26.3 *R*_J_, which also likely loses neutrals (Mogan et al., 2021) to an even more dispersed toroidal cloud. In the vicinity of Callisto's orbit, the current foreground and background removal techniques were not able to unambiguously identify a clear H_2_^+^ signal, however, future methods may help constrain the H_2_^+^ densities in this region and in the region near Europa.

These plasma measurements could directly tie into observations by the high‐energy particle detector onboard Juno, JEDI (Mauk et al., 2017), which can search for pitch‐angle depletions (e.g., Kollman et al., 2016; Lagg et al., 2003; Nénon & André, 2019) and cross‐compare with inferred neutral densities from these pickup ion observations. As the observed H_2_^+^ pickup ions likely originate from neutrals produced via radiolysis due to energetic particle bombardment and subsequent thermal desorption (e.g., Johnson et al., 2004), JEDI measurements of this charged particle environment could also be cross‐compared to these pickup observations and loss rate estimates. Constraints such as these on satellite water species loss processes and composition provide key information on the chemistry and interaction with their local charged particle environments, where present and future spacecraft will continue to shed light on these important processes over a broad range of energies (Futaana et al., 2015; Grasset et al., 2013; Paranicas et al., 2021; Phillips & Pappalardo, 2014).

Thus, the JADE observations presented here for the first time directly measure ions originating from a neutral H_2_ toroidal cloud at Jupiter, prove the cloud provides an additional plasma source in Jupiter's magnetosphere, and provide the most direct constraints on Europa's loss of neutral H_2_ via observations of the neutral toroidal cloud's primary loss process: pickup ions. Future analyses of JADE H_2_^+^ pickup ion data may further constrain loss rates and satellite‐magnetospheric interactions at Europa, Ganymede, and Callisto. These results underscore the importance of both ion composition and neutral toroidal clouds in understanding the evolution of planetary bodies, not just in the Jovian system, but at all outer planetary systems (e.g., Richardson et al., 1986; Smith & Richardson, 2021), and at exoplanetary systems.

## Supporting information

Supporting Information S1

## Data Availability

The JNO‐J/SW‐JAD‐3‐CALIBRATED‐V1.0 data presented in this manuscript and Supporting Information S1, doi:10.1007/s11214-013-9990-9, can be obtained from the Planetary Data System (PDS) at https://pds-ppi.igpp.ucla.edu/mission/JUNO/JNO/JAD.
