# Supplementary material for: Water‐Group Pickup Ions From Europa‐Genic Neutrals Orbiting Jupiter
Source: Geophys Res Lett. 2022 May 4;49(9):e2022GL098111. doi: 10.1029/2022GL098111 (PMC9286426; doi:10.1029/2022GL098111)
Supplement: Supplementary file 1 — Supporting Information S1 [file GRL-49-0-s001.pdf]

Geophysical Research Letters

Supporting Information for

H_2^+ pickup ions from Europa-genic H_2 neutrals orbiting Jupiter

J. R. Szalay[1], H. T. Smith[2], E. J. Zirnstein[1], D. J. McComas[1], L. J. Begley[1], F. Bagenal[3], P. A. Delamere[4], R. J. Wilson[3], P. Valek[5], A. R. Poppe[6], Q. Nénon[7], F. Allegrini[5,8], R. W. Ebert[5,8], S. J. Bolton[5]

[1]Department of Astrophysical Sciences, Princeton University, Princeton, New Jersey, USA

[2]The Johns Hopkins University Applied Physics Laboratory, Maryland, USA

[3]Laboratory for Atmospheric and Space Physics, University of Colorado Boulder, Boulder, Colorado, USA

[4]Geophysical Institute, University of Alaska Fairbanks, Fairbanks, AK, USA

[5]Southwest Research Institute, San Antonio, Texas, USA

[6]Space Sciences Laboratory, University of California at Berkeley, 7 Gauss Way, Berkeley, CA 94720, USA

[7]Institut de Recherche en Astrophysique et Planétologie, CNRS-UPS-CNES, Toulouse, France

[8]Department of Physics and Astronomy, University of Texas at San Antonio, San Antonio, Texas, USA

Contents of this file

Text S1 to S4
Figures S1 to S5
Table S1

Introduction

Here, we provide details of our background and foreground subtraction scheme (Text S1), calculations related to pickup ion energies (Text S2), and details on the correction factor involved in calculating numerical densities from JADE time-of-flight data (Text S3 and S4).

Text S1. Foreground and background subtraction.

We apply 3 separate subtraction methods to isolate the H_2^+ signatures in JADE's TOF by energy spectrograms on top of the existing JADE ion background subtraction routines. We must employ multiple consecutive techniques as the H_2^+ signal occurs near multiple other sources of foreground and background. Extended Data Figure 1 summarizes these methods for a period with all relevant foregrounds and backgrounds.

First we apply the nominal JADE TOF background subtraction to JADE Level 3 Version 04 data archived on the Planetary Data System (PDS). The fundamental issue is applying a so-called singles measurement from a background anode to a co-incident measurement from the sensor. We define background as signal that comes from penetrating radiation and from artifacts in time-of-flight (TOF) bins 0 – 9 (of the 93 TOF bins in Level 3 data). These signals are not affected by the JADE-I electro-optics, and can be identified by their apparent flat energy spectrum. The procedure described here will not address effects from ions that transit the instrument's electro-optics, such as false coincidences.

The nominal TOF background subtraction has two components. The first removes backgrounds from penetrating radiation. The TOF spectral shape of this penetrating radiation is found in the array N_INST_EFFECT given at the end of this section. N_INST_EFFECT is based on the normalized TOF penetrating radiation spectrum. N_INST_EFFECT is an array of length 93, with a value for each of the TOF bin (93 bins are a remapping of 248 onboard TOF channels).

The N_INST_EFFECT array is normalized and scaled by a value calculated from the background anode count rates, B_{LG}, which comes from the Level 3 Version 02 Logicals (LOG) data, also on the PDS. The scaled N_INST_EFFECT array is subtracted from the TOF data to remove backgrounds from penetrating radiation. For each TOF record, the corresponding LOG data record is found, which has 64 background counts per second measurements (one per energy step). Those 64 values are ordered in increasing count rate, then B_{LG} is calculated as the mean value of the first 8, i.e. an average of the lowest 8 count rates (not count rates of the lowest 8 energy steps), ignoring the other 56 energy steps.

There is also an instrument effect that produces background signal in TOF bins 0 – 9. These artificial TOF counts appear when there is a real signal, independent of penetrating radiation. The spectral shape of this is stored in the array LOW_TOF_ARTIFACT = [1.0, 0.4716164171695709, 0.2709026932716370, 0.1527654975652695, 0.1202179491519928, 0.1151299253106117, 0.1292860060930252, 0.2163737267255783, 0.3246990740299225, 0.3134968578815460]. This array has length 10 and is scaled so that the value in TOF bin 0 is equal to one. This correction is found by multiplying the LOW_TOF_ARTIFACT array by the count rate in TOF bin 0 after the

penetrating background has been removed. The values of the LOW_TOF_ARTIFACT array, scaled by the TOF bin 0 signal, are then subtracted from TOF bin 0 – 9. If the result for any bin is negative, the result is not used (as otherwise it would be a source of counts).

The background subtraction process is based on the background TOF spectrum in N_INST_EFFECT. This spectrum was derived from a period during Juno's first perijove, where the spacecraft passed through a relatively strong radiation environment during which JADE-I data was dominated by penetrating radiation. We use this period as a representative TOF spectrum for the penetrating radiation.

We utilize a function to scale the normalized background TOF spectrum defined in N_INST_EFFECT. It relates the signal in the background anode from the LOG dataset to the background rates for the TOF data, using the assumption that all signal in the TOF data is due to penetrating radiation during this interval. The function is defined as

$$N_{TOF} = \alpha B_{LG}^{\beta} \exp(-\gamma B_{LG})$$

The values of α and γ are updated every Juno orbit, where β=1.8 for all orbits. The values used for all intervals of this study are provided in Table S1.

B_{LG} (defined earlier) is in units of counts per second, and N_{TOF} is the background counts per second (summed over all TOF bin and averaged over all energy steps) from penetrating radiation to be subtracted from the TOF data, and is independent of energy step.

The TOF background due to penetrating radiation, P, is then $N_{TOF}S$, where S = N_INST_EFFECT. B_{LG} and hence N_{TOF} are both dependent on record t (and independent of energy step), while S is dependent on TOF bin T, hence (dependences shown in square brackets):

$$P[t,T] = N_{TOF}[t]S[T] = \alpha(B_{LG}[t])^{\beta} e^{-\gamma B_{LG}[t]} S[T]$$

The background due to the low TOF artifact effect (*LTE)* is given by the conditional equation:

$$LTE[t,E,T] = \begin{cases} \dfrac{LTA[T]}{LTA[T=0]}(D[t,E,T=0] - P[t,T=0]), & 0 \leq T \leq 9 \\ 0, & 10 \leq T \leq 92 \end{cases}$$

If $D[t,E,T=0] < P[t,T=0]$, then $LTE[t,E,T]$ would go negative, unphysically adding counts to the system. This is addressed in our final equation.

The total TOF background to be removed is then based on combining the equations for the TOF background due to penetrating radiation and the LTE. The final conditional equation describing the total TOF background is shown below:

$$TOF_{Bkgd}[t,E,T] = \begin{cases} P[t,T], & 10 \leq T \leq 92 \\ P[t,T], & 0 \leq T \leq 9 \text{ and } LTE[t,E,T] < 0 \\ LTE[t,E,T] + P[t,T], & 0 \leq T \leq 9 \text{ and } LTE[t,E,T] \geq 0 \end{cases}$$

N_INST_EFFECT=[0.019509169476715330, 0.011617905584406731, 0.0098939667442650078, 0.0082472914863843342, 0.0080060446031532638, 0.0080786954344057721, 0.0086651298559167478, 0.0094696705230461337, 0.010163735805639741, 0.0099850173264189393, 0.0039879795851712994, 0.0041290528912517246, 0.0042076746728837494, 0.0044045366428780239, 0.0043907496415554479, 0.0045886110407071089, 0.0046805329213139481, 0.0046206952881753735, 0.0042194628151709865, 0.0037766130760464737, 0.0036632419029725516, 0.0039845712742269928, 0.0043635344091353216, 0.0044573270153623187, 0.0045856127525372593, 0.0046530870516607870, 0.0048332149840258137, 0.0048161221785619898, 0.0047101314072275488, 0.0046158006473614906, 0.0045898154808399708, 0.0044183492685023361, 0.0043077457457018501, 0.0041111144143931811, 0.0040077887878444381, 0.0041506815685625363, 0.0043131529158530581, 0.0044616322511543422, 0.0041466069717306210, 0.0036710835797957304, 0.0034251726904789863, 0.0037410948917080333, 0.0041505790627431090, 0.0042201803535737150, 0.0039714761860103415, 0.0037072424229064051, 0.0038014194257417993, 0.0042125949406757570, 0.0047742999016837009, 0.0048273977935706629, 0.0044552769045095543, 0.0039654283558287684, 0.0037767924600438240, 0.0039419545776341611, 0.0042918317551884607, 0.0045834601349614996, 0.0046988814208554471, 0.0048511534620166753, 0.0047222270655204977, 0.0046313046152605510, 0.0044304961809002235, 0.0083528209779685520, 0.0098320021188703522, 0.010569042862929040, 0.0090173390270149884, 0.0092051292464298742, 0.0093959433863112004, 0.0099065493028583935, 0.0090293578031611799, 0.0083089229658671361, 0.011149788232661322, 0.010452289009846334, 0.016464574395226490, 0.017750865658724888, 0.017581449557434455, 0.018303396353314710, 0.018433501556206024, 0.033689842536383317, 0.033763210912843906, 0.033935266523295114, 0.034049073335061492, 0.033407337177810449, 0.033949104784224096, 0.033626109700132106, 0.033383171486944543, 0.033366053048017499, 0.032315217120731793, 0.030673385271360060, 0.029194691036797730, 0.027350538794182075, 0.025419769329618334, 0.024253358332026499, 0.011187484656338119]

Second, for each TOF bin, we subtract the minimum count rate (2nd panel in the top row). This represents the simplest subtraction, but is an important and effective

technique particularly in the regions explored in this study, which can have considerable fluxes of penetrating radiation that affect all energy channels equally.

Next, we account for long TOF tail due to heavy ions. Figure S2 shows a laboratory O^+ (Kim et al. 2020a) and H_2^+ response curve at 1 keV. O^+, likely the dominant heavy ion specie observed throughout the periods discussed in this study, has a long, low TOF tail that extends into the TOF range where H_2^+ is measured and has a relatively constant profile in this TOF region. We use the closest TOF range of 50-70 ns just outside the H_2^+ response as a representative sample for heavy ion backgrounds. While the O^+ response profile is relatively flat for larger TOFs up to ~90 ns, we do not extend this window to larger TOFs as this TOF region can contain contamination by higher charge states of oxygen (O^{2+}, O^{3+}, O^{4+}). We also expect the response from those additional heavy ion charge states to be relatively constant within 50-70 ns, hence, we use the nearest points along the representative O^+ response that would not incorporate counts from H_2^+.

These three methods eliminate most residual background, however, they do not account for the strong proton foreground. Due to JADE's detection mechanism, incoming protons transit through a carbon foil and exit with approximately 80% as neutral H and 20% as H^+ (Kim et al. 2020a,b). This manifests in the JADE TOF spectrogram as a forked signature for protons (Figure S3), with the lower TOF fork corresponding to the neutral H and the higher TOF fork due to the H^+ (Kim et al. 2020). The higher TOF fork occurs at very similar locations to where H_2^+ counts are registered.

To subtract the H^+ forked signature, we use an in-flight reference H^+ TOF spectra. The ideal proton reference spectra should exhibit a clean proton signal across all energies, no H_2^+ signature, and minimal additional low-TOF contributions from heavy ions. There was a unique event during Juno's 12th perijove where the spacecraft flew through auroral field lines connected to Io's Main Alfvén Wing (Szalay et al. 2021). During this period, from 2018-091 9:19:00 to 9:21:20, protons were observed and energized throughout a large energy range across multiple Juno instruments (Sulaiman et al. 2020; Clark et al. 2020) and JADE recorded very large proton fluxes across its entire energy range. Additionally, due to the position of Juno at that time, there were very few heavy ions detected, and no observable signature of H_2^+ is present. This observation provides a near-perfect in-flight proton reference TOF signature, as shown in Figure S3 normalized at each energy to the first proton peak.

Since the first fork of any spectrogram following the AMU/Q = 1 line is free of H_2^+, we fit the peak of the first fork in the data to derive the properties of the foreground H^+ distribution. Using that, we can predict the shape of the 2nd fork of the spectrogram because we know the percentage of H^+ that is neutralized in the foils from our reference proton spectra. A synthetic signal of H^+ can be generated, which is then subtracted from any overlaying H_2^+ signal in the 2nd fork, along the M/Q=2 curve. We create an isolated H^+ TOF spectrogram corresponding to each dataset, shown in the middle row of Figure S1, which is slightly different in each of the five panels as the values are scaled to the

peak proton signatures in the first row. In the final step, we subtract this H^+ signature from the data, and remove all counts that correspond to AMU/Q ≤ 1.5 and AMU/Q ≥ 2.5, shown in the last column of Figure S1.

Text S2. Pickup ion energy.

Pickup ions are injected at a velocity in the corotating frame of $\boldsymbol{v}_{PUI,cor} = \boldsymbol{v}_{cor} - \boldsymbol{v}_{orb}$, where $v_{cor} = \omega r \cos\theta$ is the corotational speed, $v_{orb} = \sqrt{\mu/r}$ is the orbital speed, $\omega = 1.757 \times 10^{-4}$ s^{-1} is Jupiter's angular rotation frequency (period of 9.93 hr), $\mu = 1.267 \times 10^{17}$ m^3 s^{-1} is Jupiter's standard gravitational parameter, and θ is latitude. In a reference frame centered on Jupiter but not rotating with the planet, PUIs have a speed in the range of $v_{PUI,inj} = |2\boldsymbol{v}_{cor} - \boldsymbol{v}_{orb}|$ to $v_{PUI,min} = v_{orb}$. Juno's relative motion plays a role in the detected PUI energies, hence the expected PUI injection speed in the spacecraft frame for PUIs is $v_{PUI,Juno} = \left|\boldsymbol{v}_{PUI,inj} - \boldsymbol{v}_{Juno}\right| = \left|\boldsymbol{v}_{Juno} - 2\boldsymbol{v}_{cor} + \boldsymbol{v}_{orb}\right|$, where $\boldsymbol{v}_{Juno}$ is the velocity vector of the Juno spacecraft with respect to Jupiter's center in a non-rotating frame.

Text S3. TOF Count Rate Correction Factor.

The JADE TOF data produce sums counts from all anodes 0-11 and does not track which anode counts were observed on. Due to the configuration of JADE and mounting on the spacecraft with respect to the Juno spin vector, anodes 0-3 view the same portion of the sky as anodes 4-7 each half spin as shown in Figure S4. As all calculations in this study are performed on data averaged over many spins, any counts measured on anodes 0-7 are double-counted and the count rates must be normalized to account for this. To be consistent with the numerical densities calculated below 10 keV/Q, we use count rates below 10 keV/Q to determine the correction factor.

Let R_A represent the spin-averaged count rates observed on anodes 4-7 (equivalent to spin-averaged rates on anodes 0-3) and R_B represent the count rates observed on anodes 8-11, all below 10 keV/Q. The observed TOF count rates are given by $R_{obs} = 2R_A + R_B$. The "true" full-sky average count rates are given by $R = R_A + R_B$. Taking the ratio R_{obs}/R of these two values, $R = \eta R_{obs}$ where

$$\eta = \frac{R_A + R_B}{2R_A + R_B}$$

We can estimate η using a different JADE data product ("species" data) that records count rates as a function of look direction and energy for various compositional groups. Specifically, we use the data product that sums counts within TOF space for H^+ and partially captures counts from H_2^+ as well (species=3 in the JADE data files). Given that the H_2^+/H^+ is ~8% during all periods, this product effectively allows us to determine the directionality of protons. Assuming both H^+ and H_2^+ are observed from similar directions, i.e. local corotation velocity at the Juno spacecraft, we use the species data to determine

R_A and R_B, allowing for an estimate of η. For all but one period, count rates are observed to peak in anodes 6-7, hence the majority of spin-averaged TOF count rates are too large by approximately a factor of 2. Table S1 gives η for each period, where $\eta \approx 0.5$ for all but the first period.

Text S4. TOF Density Correction Factor.

As described in the text, there is an overlap in TOF x E space between the secondary H^+ "fork" and AMU/Q=2 line below ~1 keV. If there are significantly higher densities of H^+ compared to H_2^+, as is the case in these periods investigated here, the H^+ foreground subtraction method can over-subtract a fraction of H_2^+ counts in the vicinity of the H^+ fork. This leads to an underestimate of the total H_2^+ density, where a scale factor needs to be applied such that $n = \varepsilon n_{num}$, where n_{num} is the numerical number density we calculate, and ε is the scaling factor to calculate the "true" number density n. To assess the extent to which our method underestimates H_2^+ densities, we leverage a period when Juno transited Ganymede's plasma wake during PJ34 and observed the largest fluxes of H_2^+ up to this time.

Figure S5a shows a TOF spectra from 2021-158 16:46:46 to 16:47:45, where H_2^+ count rates are considerably larger than those from the H^+ fork. We choose this period as the H_2^+ signature has a similar energy distribution to those investigated in this study. For this period, the H_2^+ signature after removal of the H^+ counts in Figure S5b exhibits counts on lower and higher TOF values near the AMU/Q=2 line, where the peak count rates occur at slightly higher TOFs than the nominal AMU/Q=2 line. This can be compared with Figure S5e & f, which shows the TOF spectra from Figure 1 corresponding to the spatial region of 14-15 R_J. In Figure S5f, the H_2^+ signature is similar to that from S5b, however, there is a depletion of counts between the H^+ fork and AMU/Q=2 line, due to slight over-subtraction of H^+ count rates.

To simulate and quantify how over-subtraction of a similar H_2^+ distribution leads to changes in the density estimates, we incrementally increased the TOF values for the H^+ fork and subtracted all count rates for TOF values inside this enhanced H^+ fork. This is done to determine an upper bound to the over-subtraction that occurs when removing counts from the H^+ fork. We calculated numerical densities for these over-subtracted TOF spectra and compared them to those from the "true" density from Figure S5b. Figure S5c & d shows modified versions of the count rates in Figure S5b with TOF values from the H^+ fork times 1.02 and 1.05, which leads to underestimated densities of 84% (ε=1.2) and 63% (ε=1.6) of the true density.

The case shown in Figure S5d for ε=1.6 produces more apparent over-subtraction than the example data in Figure S5f (and all other periods in Figure 1), where the large degree of over-subtraction leads to less available pixels with count rates inside the AMU/Q=2 line than those in Figure 1. Hence, this case represents a conservative upper bound and

we expect ε is no larger than 1.6. Likely, it is closer to that shown in Figure S5c corresponding to ε=1.2 as this more closely resembles the TOF spectra shown in Figure 1.

For the densities and mass loss calculations in this paper, we therefore use a range of ε=1.0, corresponding to no over-subtraction, to ε=1.6, a conservative upper bound on the correction factor. Figure 2 shows densities corresponding to a correction factor of ε=1.2 and the total H_2^+ mass loss of 1.2 ± 0.7 kg s^{-1} is calculated using the full range from ε=1.0-1.6.

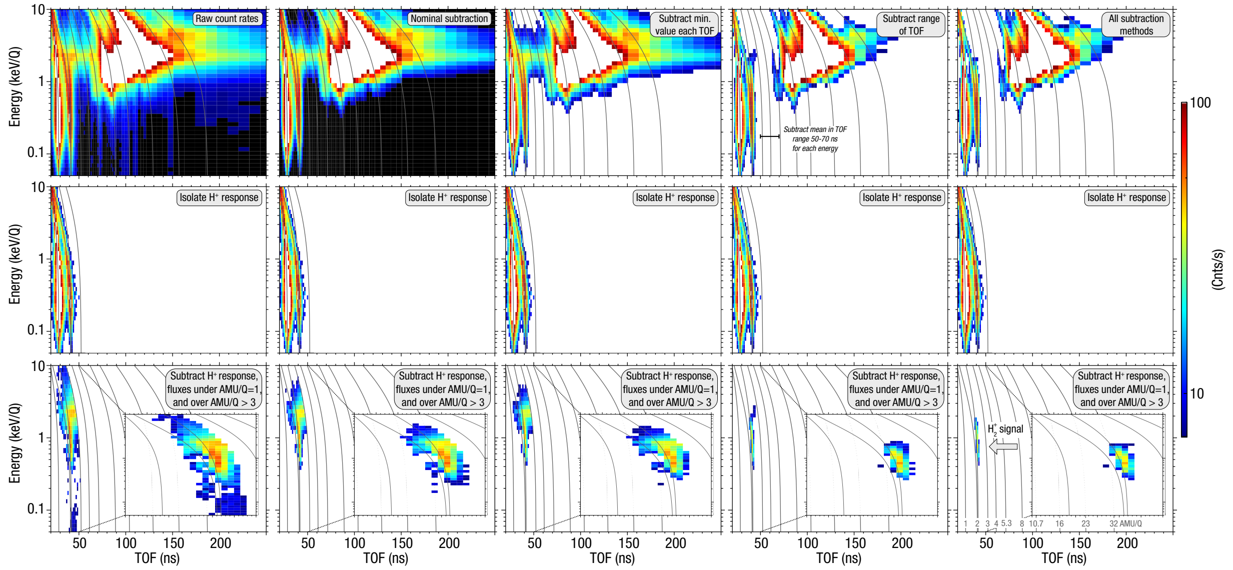

Figure S1. Background subtraction methods for an example period of H_2^+ observations from 2020-100T23:00 to 2020-101T00:30. The top row shows the various combinations of background subtraction applied to the full TOF dataset. The middle row shows the isolated H^+ feature, scaled to each TOF observation in the top row. The bottom row shows the derived H_2^+ rates in the top row after subtracting the middle row and removing counts below an AMU/Q value of 1.5 and above an AMU/Q value of 2.5.

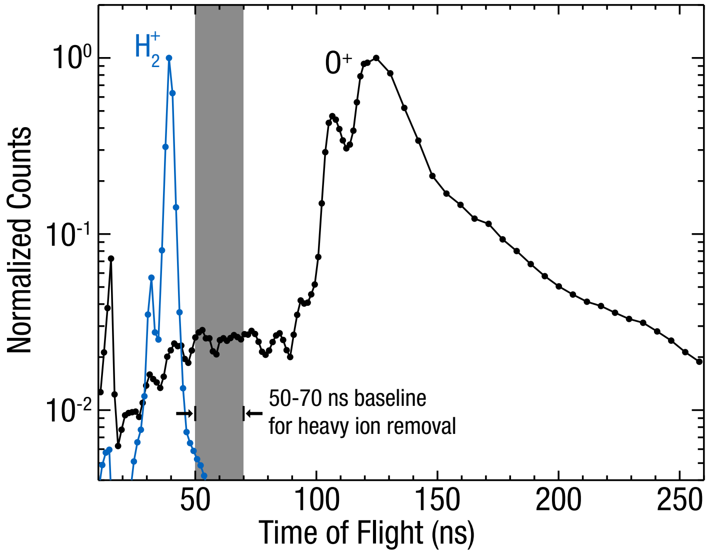

Figure S2. JADE H_2^+ and O^+ response function at 1 keV/Q from laboratory calibrations. The window used to determine the baseline heavy ion backgrounds of 50-70 ns is chosen to be the closest portion of the heavy ion low-TOF tail immediately adjacent to the H_2^+ response curve.

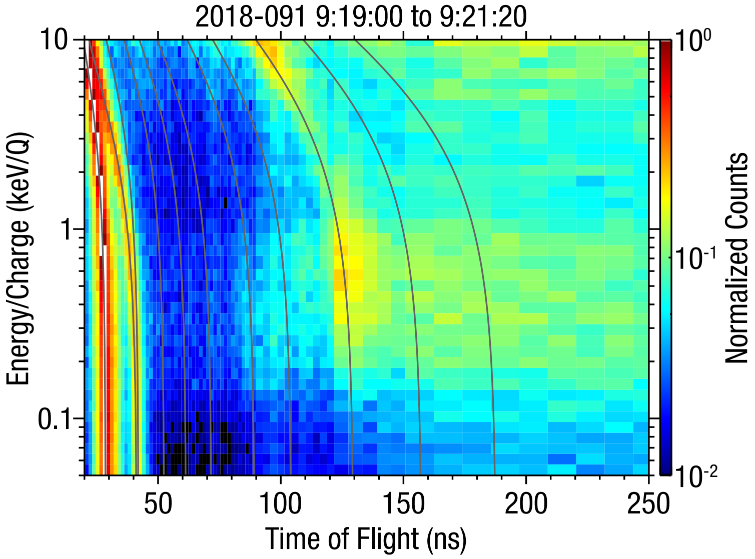

Figure S3. Reference H^+ TOF spectrogram from 2018-091 9:19 to 9:21:20. This measurement occurred when JADE was connected to Io's Main Alfvén Wing.

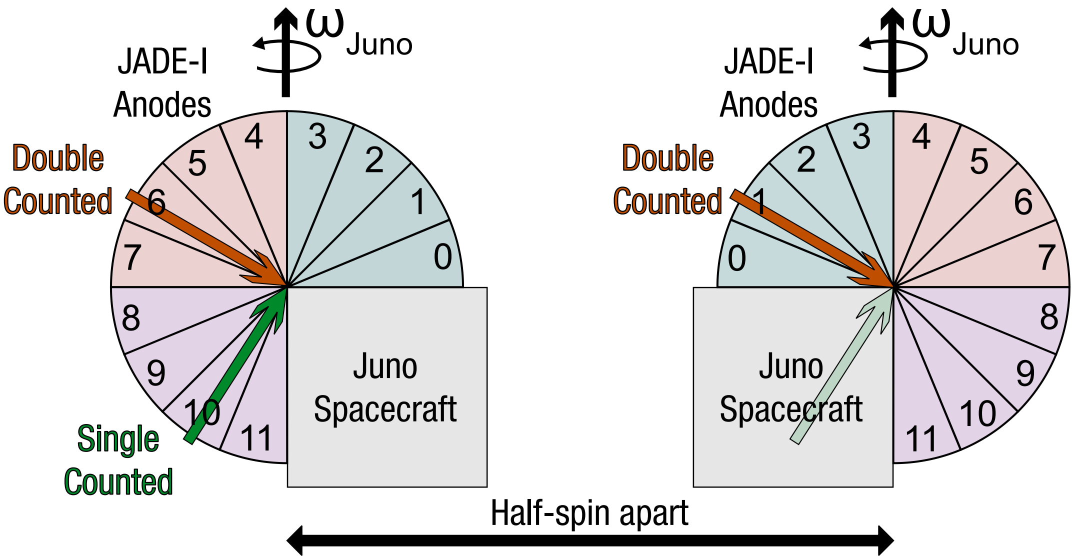

Figure S4. Configuration and mounting of the JADE instrument onboard Juno. For the TOF data product which does not track anodes, counts observed in anodes 0-7 are double counted each spin, while counts observed in anodes 8-11 are not.

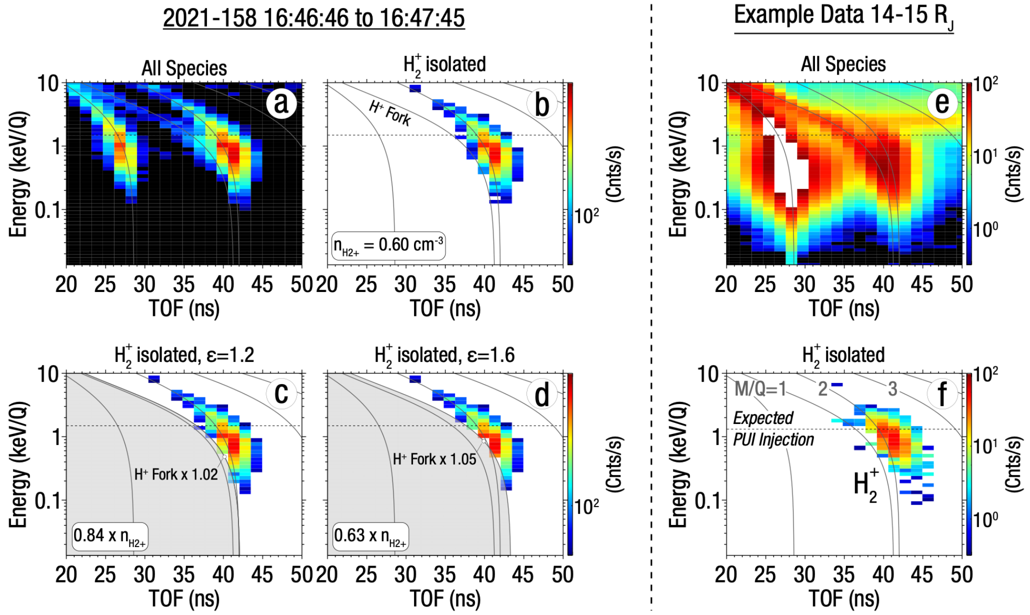

Figure S5. TOF spectra from 2021-158 16:46:46 to 16:47:45 where JADE observed prominent H_2^+ ions in the vicinity of Ganymede for all species (a) and H_2^+ isolated (b). Different proton over-subtraction schemes are shown in (c) and (d). Panels (e) and (f) show the same data from Figure 1 from 14-15 R_J.

Period	Orbit	Start	Stop	ρ_{min} (R_J)	ρ_{max} (R_J)	η	α (unitless)	γ (s/count)
**1**	24	2019-360T02:48:12	2019-360T03:08:12	14.7	14.9	0.83	7.1929×10^{-5}	7.1991×10^{-6}
**2**	26	2020-100T19:05:24	2020-100T20:20:24	16.7	17.5	0.53	7.0487×10^{-5}	7.0275×10^{-6}
**3**	26	2020-100T22:55:24	2020-101T00:07:47	14.1	15.0	0.52	7.0487×10^{-5}	7.0275×10^{-6}
**4**	29	2020-259T08:57:48	2020-259T09:57:48	15.9	16.6	0.56	7.8410×10^{-5}	7.6716×10^{-6}
**5**	31	2020-365T05:49:24	2020-365T06:54:24	15.0	15.7	0.56	8.4951×10^{-5}	7.8349×10^{-6}
**6**	32	2021-051T23:06:37	2021-052T00:22:48	16.6	17.4	0.54	9.1211×10^{-5}	8.3077×10^{-6}
**7**	32	2021-052T03:52:48	2021-052T05:07:48	13.3	14.2	0.53	9.1211×10^{-5}	8.3077×10^{-6}
**8**	33	2021-105T04:09:25	2021-105T04:19:25	17.8	18.0	0.56	8.9203×10^{-5}	8.2352×10^{-6}
**9**	33	2021-105T07:39:25	2021-105T08:59:25	14.8	15.7	0.54	8.9203×10^{-5}	8.2352×10^{-6}
**10**	36	2021-245T07:37:58	2021-245T08:42:58	14.3	15.1	0.53	9.3578×10^{-5}	8.4369×10^{-6}
**11**	37	2021-288T21:40:26	2021-288T22:50:26	17.2	18.0	0.55	1.0233×10^{-4}	9.0081×10^{-6}
**12**	37	2021-289T03:33:10	2021-289T04:43:10	13.3	14.1	0.53	1.0233×10^{-4}	9.0081×10^{-6}

Table S1. Time ranges, distances, and background subtraction coefficients for all data used in the analysis throughout this manuscript where Juno was within 1 R_J from the magnetic equator and H_2+ could be unambiguously identified.
